# Supplementary material for: Nucleotide context models outperform protein language models for predicting antibody affinity maturation
Source: bioRxiv. 2025 Jun 18:2025.06.16.659977. Preprint. [Version 1] doi: 10.1101/2025.06.16.659977 (PMC12262217; doi:10.1101/2025.06.16.659977)
Supplement: Supplementary file 1 [file media-1.pdf]

Supplementary Materials

**Data set**	**Species**	**PCPs**	**Clonal families**	**Median PCPs/CF**	**Median subs./PCP**	**Comments**
Tang et al. IgH [48]	Human	438,302	130,621	2	2	In OAS (AbLang training set)
Jaffe et al. IgH [31]	Human	176,656	49,430	2	4	In OAS (AbLang training set) and Thrifty-prod training set
Rodriguez et al. IgH [30]	Human	18,639	6,837	2	4	
Ford et al. IgH [29]	Human	2,407	1,182	2	3	
Replay IgH [32]	Mouse	5,378	129	44	1	
Replay Igκ [32]	Mouse	5,176	129	40	1	

Table S1: Summary of data sets used. All human data results in the main text are based on the Tang et al. IgH data set. tab:pcp stats

Model	Overlap	R-prec.	Sub. Acc.	CSP Perp.
S5F	0.771	0.111	0.277	7.38
S5F + ESM-1v	0.595	0.099	0.341	8.10
S5F + BLOSUM62	0.780	0.113	0.319	7.01
Thrifty-SHM	0.818	0.147	0.347	6.22
Thrifty-SHM + ESM-1v	0.603	0.107	0.345	7.73
Thrifty-SHM + BLOSUM62	0.823	0.150	0.369	6.00
Thrifty-prod	**0.907**	**0.164**	0.393	**5.66**
ESM-1v	0.710	0.073	0.258	12.43
AbLang2	0.754	0.086	**0.41**	6.23

Table S2: Model performance on Jaffe et al. IgH data set. The best model for each metric is indicated in **bold**. tab:jaffe˙results

Model	Overlap	R-prec.	Sub. Acc.	CSP Perp.
S5F	0.782	0.118	0.263	8.20
S5F + ESM-1v	0.617	0.112	0.328	9.12
S5F + BLOSUM62	0.795	0.122	0.300	7.84
Thrifty-SHM	0.823	0.145	0.326	6.87
Thrifty-SHM + ESM-1v	0.624	0.116	0.334	8.63
Thrifty-SHM + BLOSUM62	0.835	0.149	0.350	6.67
Thrifty-prod	**0.900**	**0.163**	**0.370**	**6.36**
ESM-1v	0.739	0.091	0.252	13.48
AbLang2	0.780	0.095	0.353	8.20

Table S3: Model performance on Rodriguez et al. IgH data set. The best model for each metric is indicated in **bold**. tab:rodriguez˙results

Model	Overlap	R-prec.	Sub. Acc.	CSP Perp.
S5F	0.779	0.106	0.261	8.33
S5F + ESM-1v	0.613	0.096	0.324	9.61
S5F + BLOSUM62	0.790	0.107	0.300	8.01
Thrifty-SHM	0.824	0.128	0.324	7.00
Thrifty-SHM + ESM-1v	0.620	0.102	0.328	8.99
Thrifty-SHM + BLOSUM62	0.834	0.132	0.345	6.80
Thrifty-prod	**0.900**	**0.141**	**0.365**	**6.52**
ESM-1v	0.733	0.082	0.249	13.72
AbLang2	0.787	0.090	0.332	8.61

Table S4: Model performance on Ford et al. IgH data set. The best model for each metric is indicated in **bold**. tab:ford˙results

Model	Modeling perspective	Biological process	EPAM framework	Species evaluated in EPAM
Thrifty-SHM [16]	ME	SHM	NT	Human
Thrifty-prod [16]	ME	Implicit SHM-selection	NT	Human
S5F [13]	ME	SHM	NT	Human
S5F + ESM-1v	ME+LM	Explicit SHM-selection	NT-AA	Human
S5F + BLOSUM62 [54]	ME	Explicit SHM-selection	NT-AA	Human
Thrifty-SHM + ESM-1v	ME+LM	Explicit SHM-selection	NT-AA	Human
Thrifty-SHM + BLOSUM62	ME	Explicit SHM-selection	NT-AA	Human
ESM-1v (model 1) [35]	LM	Selection	AA	Human, mouse
AbLang [21, 28]	LM	Implicit SHM-selection	AA	Human, mouse
ReplaySHM [32]	ME	SHM	NT	Mouse
ReplaySHM + DMS	ME	Explicit SHM-selection	NT-AA	Mouse
ReplaySHM + BLOSUM62	ME	Explicit SHM-selection	NT-AA	Mouse
ReplaySHM + ESM-1v	ME+LM	Explicit SHM-selection	NT-AA	Mouse

Table S5: Classifications for all models considered. Models are characterized by the modeling perspective considered: molecular evolution (ME) or language modeling (LM). The molecular evolution perspective includes mutation models (SHM) and models that combine mutation and selection (explicit SHM-selection), while language models either characterize the joint substitution process (implicit SHM-selection) or selection alone. EPAM frameworks are classified by the input data type, considering nucleotide (NT) and/or amino acid (AA) sequences. Models are evaluated on human and mouse data sets in EPAM where applicable.

tab:model˙classifications

Model	Overlap	R-prec.	Sub. Acc.	CSP Perp.
S5F	0.783	0.096	0.262	8.19
S5F + ESM-1v	0.616	0.100	0.326	9.11
S5F + BLOSUM62	0.786	0.098	0.299	7.92
Thrifty-SHM	0.834	0.127	0.328	6.86
Thrifty-SHM + ESM-1v	0.627	0.103	0.331	8.65
Thrifty-SHM + BLOSUM62	0.836	0.129	0.344	6.73
Thrifty-prod	**0.923**	**0.139**	**0.363**	**6.46**
ESM-1v	0.744	0.082	0.255	12.61
AbLang2	0.774	0.097	0.356	7.61

Table S6: Model performance on the Tang et al. IgH data set. The best model for each metric is indicated in **bold**. tab:tang˙results

	**23**	**41**	**43**	**98**	**102**	**104**	**118**
Observed	0.14	0.15	0.13	0.19	0.25	0	0
S5F	0.77	0.46	0.33	0.60	0.98	0.72	0.43
Thrifty-SHM	0.54	0.51	0.39	0.49	0.85	0.61	0.43
ESM-1v	0.11	0.11	0.15	0.11	0.14	0.11	0.16
Thrifty-SHM + ESM-1v	0.09	0.09	0.10	0.10	0.11	0.09	0.10
AbLang2	0.12	0.27	0.25	0.30	0.46	0.12	0.39
Thrifty-prod	0.27	0.27	0.22	0.23	0.49	0.27	0.23

Table S7: Quantile of the observed or expected number of substitutions at known conserved sites in the Tang et al. IgH data set. tab:conserved˙sites˙quantile

Model	Overlap	R-prec.	Sub. Acc.	CSP Perp.
ReplaySHM	0.790	0.143	0.416	5.07
ReplaySHM + BLOSUM62	0.777	0.129	0.420	**5.01**
ReplaySHM + DMS	**0.820**	**0.146**	**0.430**	5.10
ReplaySHM + ESM-1v	0.535	0.034	0.374	7.69
ESM-1v	0.530	0.011	0.212	15.9
AbLang2	0.623	0.023	0.385	5.88

Table S8: Model performance on Replay IgH data set. The best model for each metric is indicated in **bold**. tab:gcreplay˙igh˙results

Model	Overlap	R-prec.	Sub. Acc.	CSP Perp.
ReplaySHM	0.717	0.090	0.386	4.91
ReplaySHM + BLOSUM62	0.710	0.091	0.419	4.79
ReplaySHM + DMS	**0.786**	**0.125**	**0.423**	**4.77**
ReplaySHM + ESM-1v	0.550	0.039	0.377	8.70
ESM-1v	0.547	0.017	0.182	21.24
AbLang2	0.646	0.022	0.367	7.30

Table S9: Model performance on Replay Igκ data set. The best model for each metric is indicated in **bold**. tab:gcreplay˙igk˙results

Model	IgH aff.	IgK aff.	IgH expr.	IgK expr.
ESM-1v 1	0.0317	0.2636	0.3701	0.5538
ESM-1v 2	0.0466	0.2677	0.4016	0.5739
ESM-1v 3	0.0438	0.2414	0.3890	0.5284
ESM-1v 4	0.0527	0.2427	0.4163	0.5688
ESM-1v 5	0.0421	0.2551	0.3788	0.5699
ESM-1v ensemble	0.0477	0.2658	0.4076	0.5793

Table S10: DMS and ESM-1v correlations for Replay IgH and IgK naive sequences. The ESM-1v values used here are the log transformed probability ratios (site-specific probabilities relative to the wildtype amino acid). Correlation values represent the Spearman rank correlation coefficient between DMS affinity or expression measures and ESM-1v predictions. We observed a stronger correlation between ESM-1v and DMS expression values relative to affinity. Similar trends were found in large benchmarks of DMS measurements and ESM models [39]. tab:model˙affinities

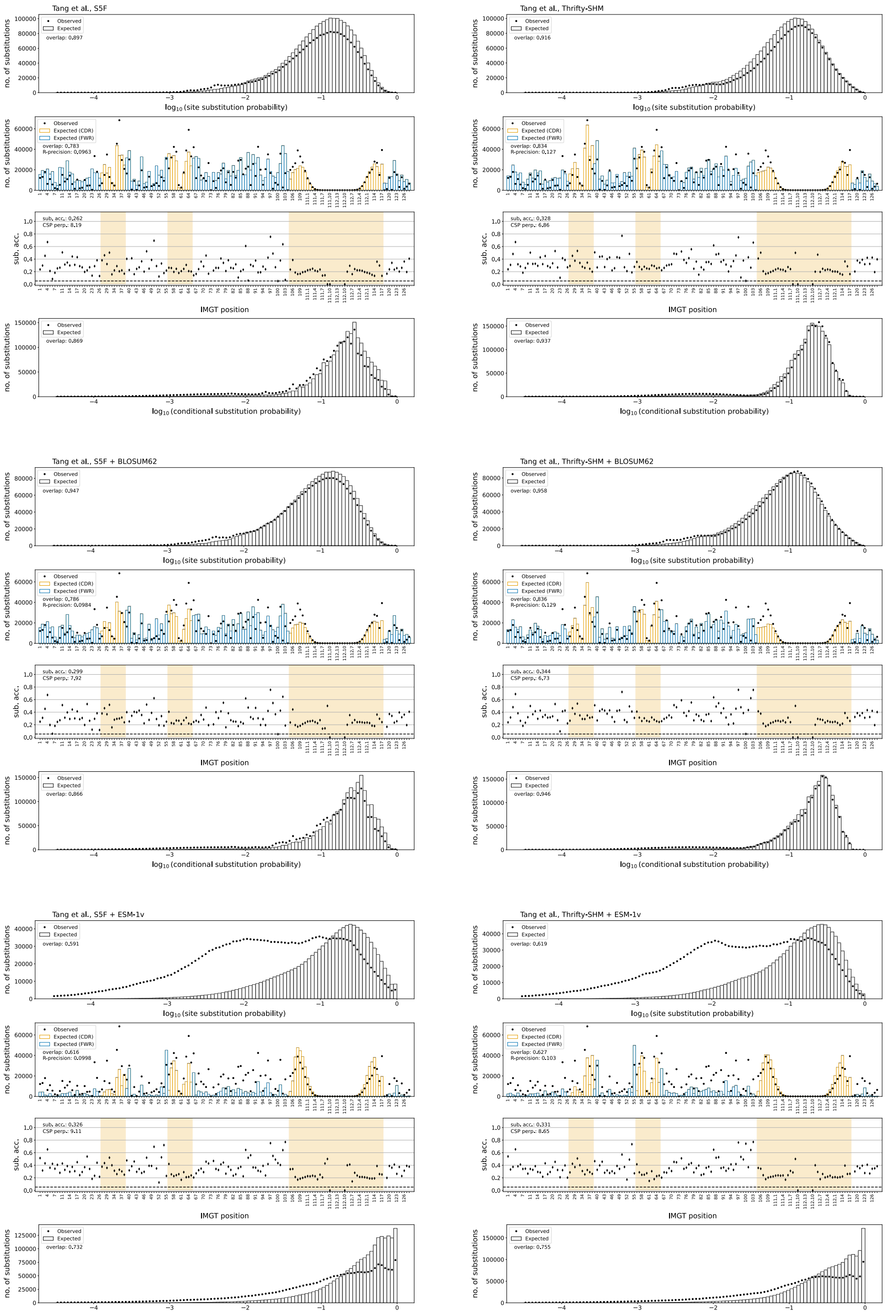

Figure S1: NT and NT-AA framework models on the Tang et al. data set. For each model, there are four panels. The top panel shows the observed and expected number of substitutions where the sites are binned according to their predicted SSP. The second panel shows the observed and expected number of substitutions over site position. The third panel shows the per-site substitution accuracy. The bottom panel shows the observed and expected number of substitutions at sites of substitution, and the sites are binned according to their predicted CSP. The hatch-filled bands are the standard deviations on the expectations.

fig:tang˙all˙1

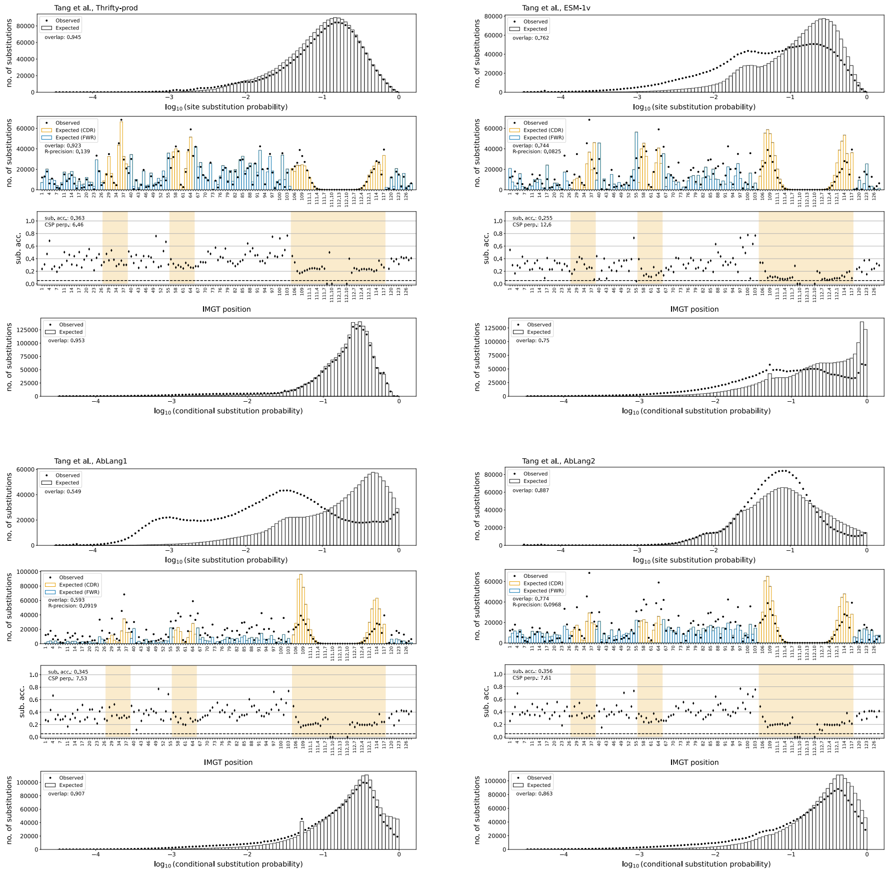

Figure S2: AA framework models on the Tang et al. data set. For each model, there are four panels. The top panel shows the observed and expected number of substitutions where the sites are binned according to their predicted SSP. The second panel shows the observed and expected number of substitutions over site position. The third panel shows the per-site substitution accuracy. The bottom panel shows the observed and expected number of substitutions at sites of substitution, and the sites are binned according to their predicted CSP. The hatch-filled bands are the standard deviations on the expectations. fig:tang˙all˙2

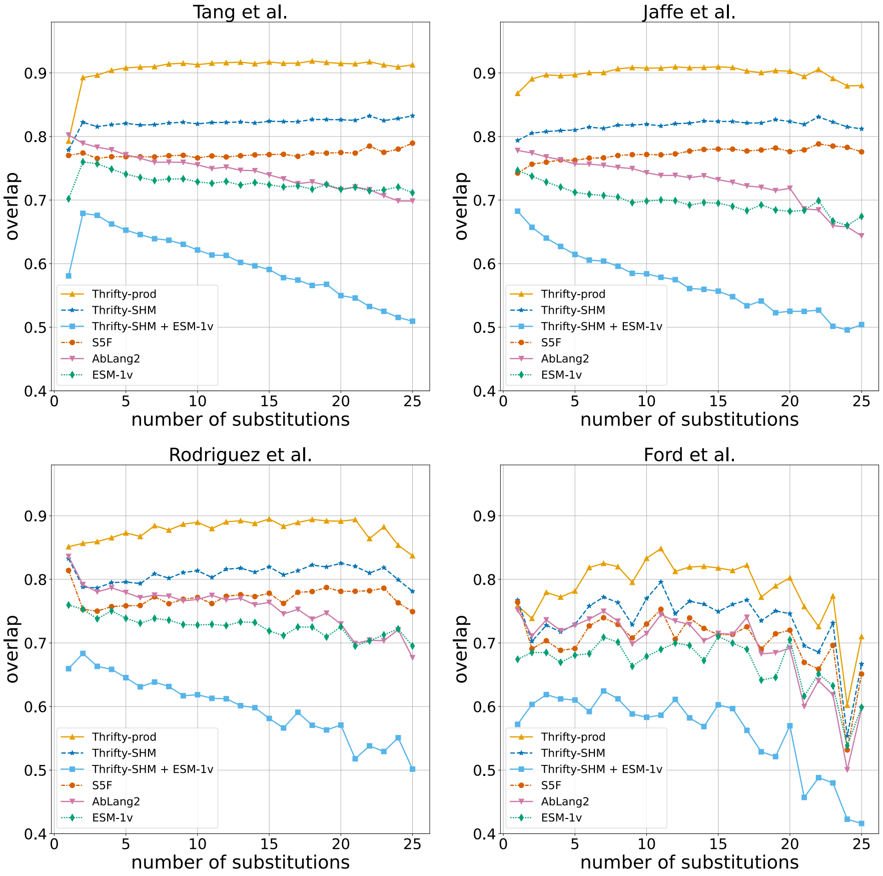

Figure S3: Overlap stratified by number of substitutions per PCP evaluated on the human data sets. fig:human˙overlap˙vs˙nmuts

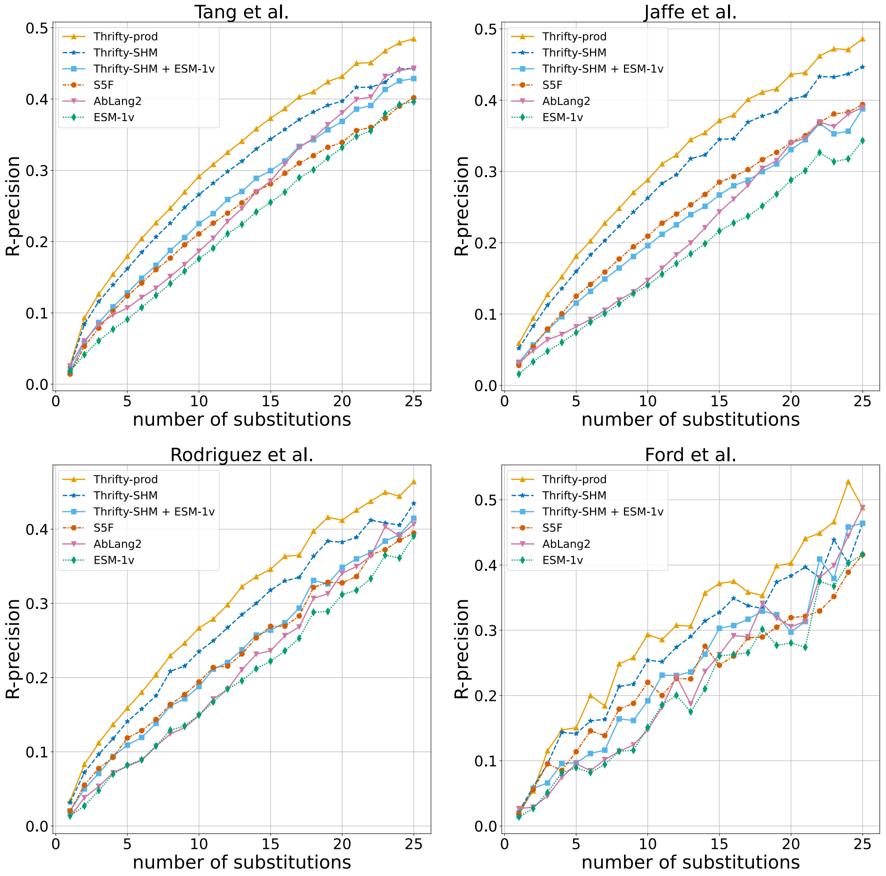

Figure S4: R-precision stratified by number of substitutions per PCP evaluated on the human data sets.

fig:human˙rprec˙vs˙nmuts

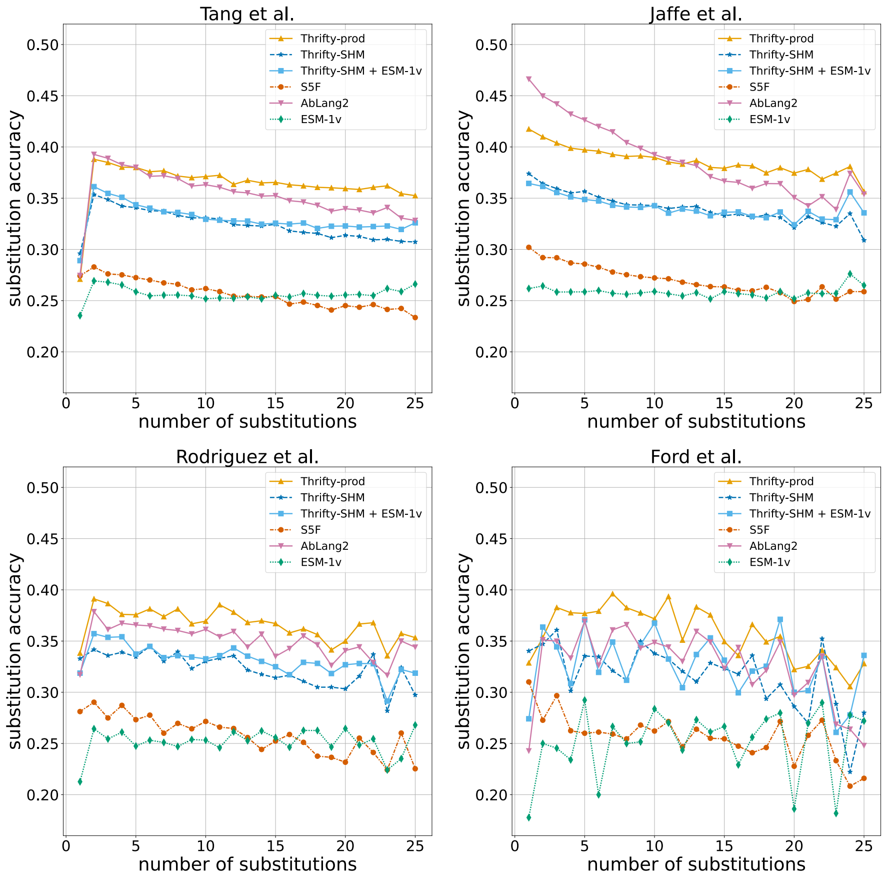

Figure S5: Substitution accuracy stratified by number of substitutions per PCP evaluated on the human data sets. fig:human`subacc`vs`nmuts

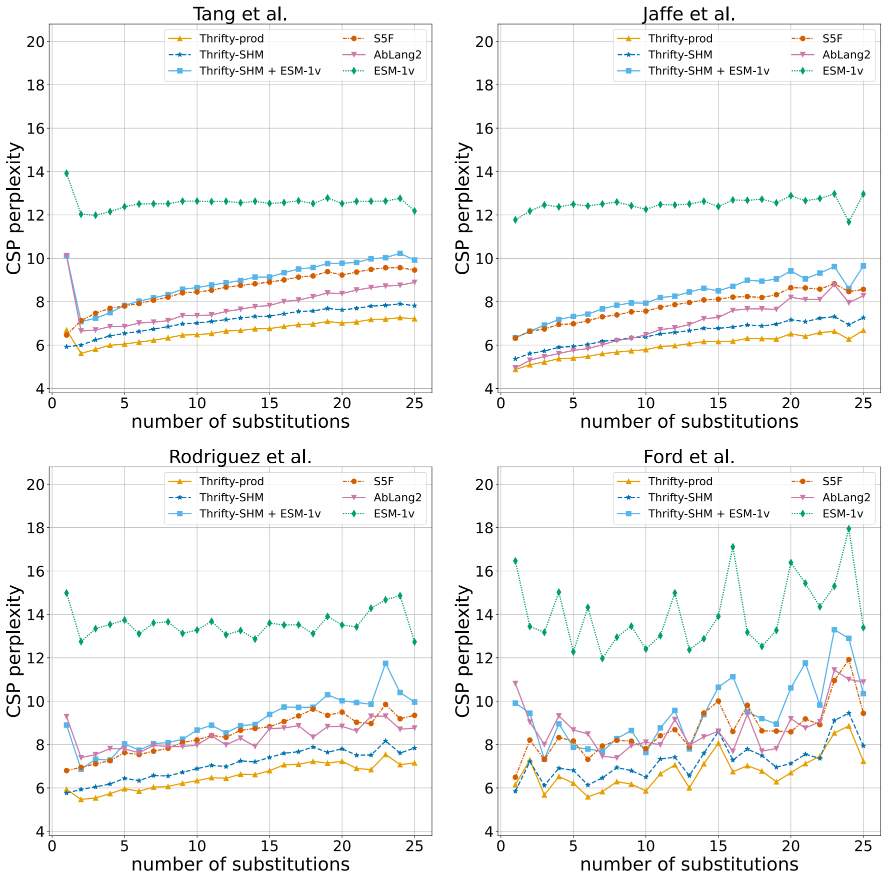

Figure S6: CSP perplexity stratified by number of substitutions per PCP evaluated on the human data sets. fig:human˙cspperp˙vs˙nmuts

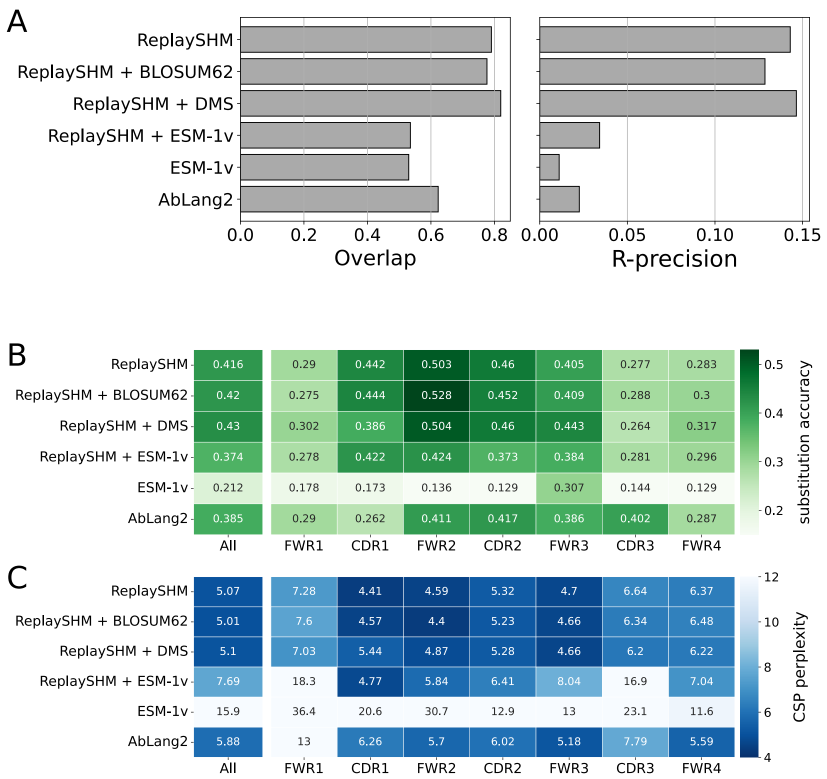

Figure S7: Performance of models on Replay heavy chain data. (A) Site substitution overlap and R-precision. (B) Substitution accuracy of the most probable amino acid at sites of substitution. (C) CSP perplexity. fig:gcreplay˙igh

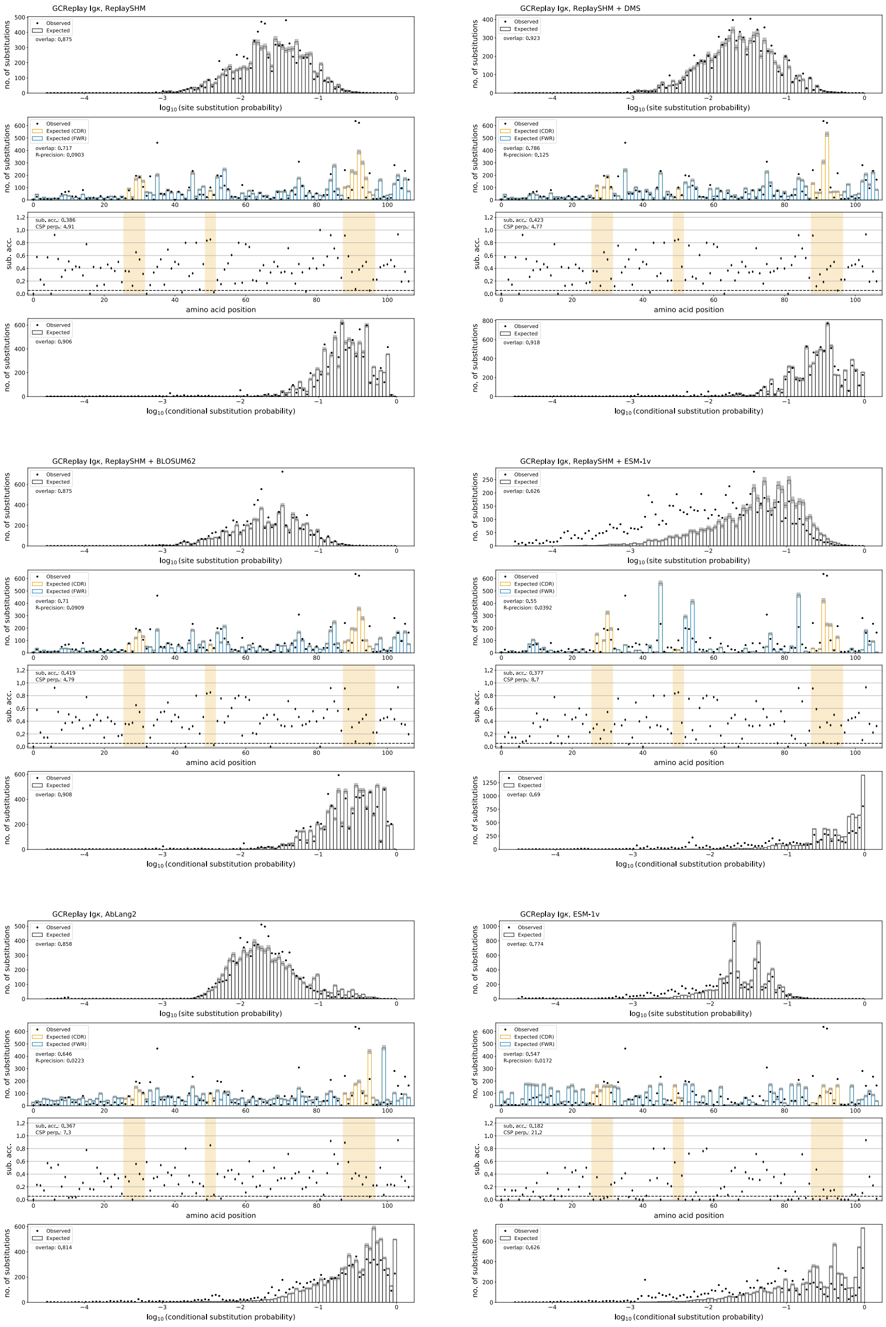

Figure S8: All models on Replay light chain data set. For each model, there are four panels. The top panel shows the observed and expected number of substitutions where the sites are binned according to their predicted SSP. The second panel shows the observed and expected number of substitutions over site position. The third panel shows the per-site substitution accuracy. The bottom panel shows the observed and expected number of substitutions at sites of substitution, and the sites are binned according to their predicted CSP. The hatch-filled bands are the standard deviations on the expectations. fig:gcreplay˙igk˙all

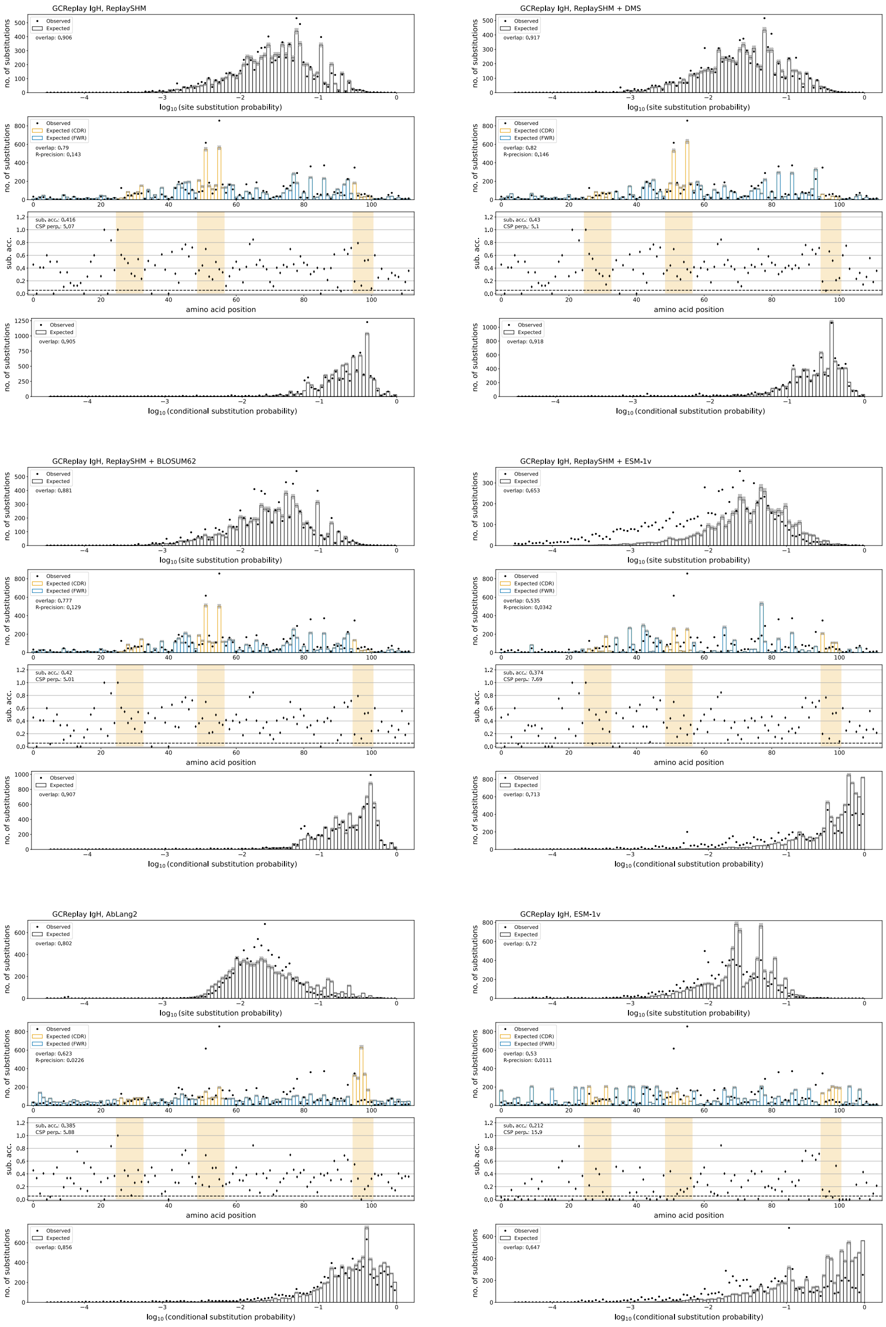

Figure S9: All models on Replay heavy chain data set. For each model, there are four panels. The top panel shows the observed and expected number of substitutions where the sites are binned according to their predicted SSP. The second panel shows the observed and expected number of substitutions over site position. The third panel shows the per-site substitution accuracy. The bottom panel shows the observed and expected number of substitutions at sites of substitution, and the sites are binned according to their predicted CSP. The hatch-filled bands are the standard deviations on the expectations. fig:gcreplay˙igh˙all

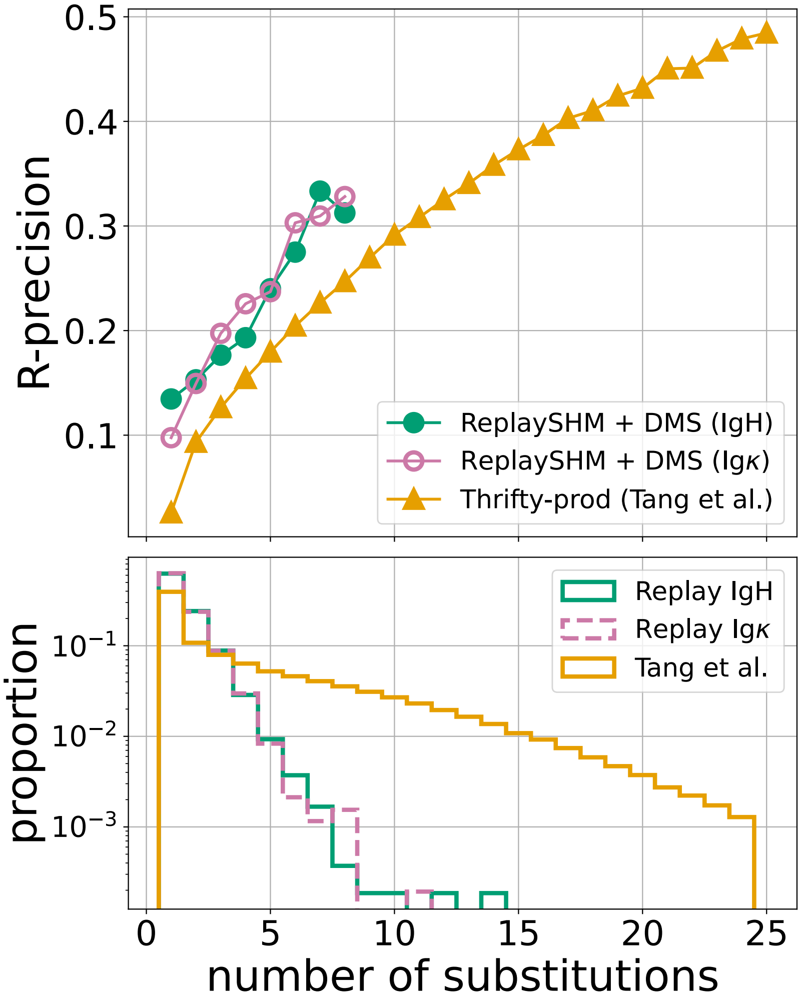

Figure S10: (Upper): When stratified by number of substitutions per PCP, R-precision of ReplaySHM + DMS on Replay data is better than models on human repertoire data. The Thrifty-prod model is shown, which is the best model that we evaluated on human data. (Lower): Distribution of the number of substitutions per PCP in the Replay data sets and the Tang et al. data set. The Tang et al. data (and similarly in the other human repertoire data sets) consists of more PCPs with higher number of substitutions than the Replay data. fig:rprec˙vs˙nmuts

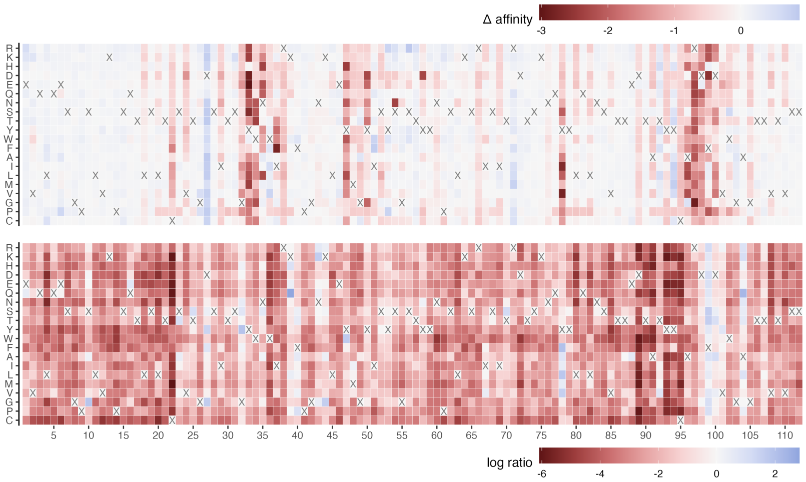

Figure S11: ESM-1v predictions do not agree with DMS affinity findings in Replay IgH naive sequence. (Upper): DMS affinity measurements for the Replay IgH naive sequence. (Lower): ESM-1v predictions for the same naive sequence, presented as the log transformed probability ratio. The wildtype amino acid at each site in the naive sequence is depicted with a 'X'. Values are relative to the wildtype amino acid in the naive sequence context. fig:sel˙heatmaps

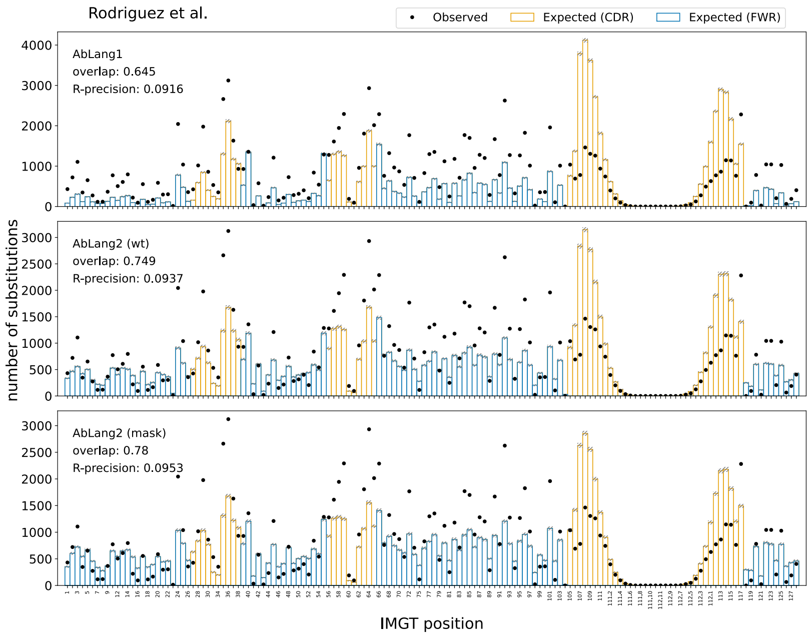

Figure S12: Improved antibody prediction with AbLang2 on Rodriguez et al. IgH data set. Results are presented for AbLang1 and AbLang2 using both scoring strategies. AbLang2 (mask) achieved the best performance across all metrics, and was used in all other AbLang2 results presented. fig:ablangs˙comp

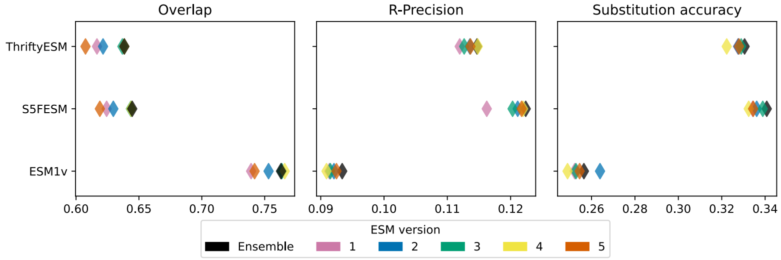

Figure S13: The ensemble ESM-1v model does not offer a significant performance improvement over individual models in the Rodriguez et al. data set. For reference, the best-performing model on this data set obtained an overlap of 0.900, R-precision of 0.163, and substitution accuracy of 0.370. This trend was observed in when ESM-1v was used in a standalone model as well as when it was used to compute selection factors in the NT-AA framework. No model consistently performed the best across all metrics. Model 1 of the ESM-1v ensembles was used in all other ESM-1v results presented. fig:esm˙models
